# In situ brain tumor detection using a Raman spectroscopy system—results of a multicenter study

**DOI:** 10.1038/s41598-024-62543-9

**Published:** 2024-06-10

**Authors:** Katherine Ember, Frédérick Dallaire, Arthur Plante, Guillaume Sheehy, Marie-Christine Guiot, Rajeev Agarwal, Rajeev Yadav, Alice Douet, Juliette Selb, Jean Philippe Tremblay, Alex Dupuis, Eric Marple, Kirk Urmey, Caroline Rizea, Armand Harb, Lily McCarthy, Alexander Schupper, Melissa Umphlett, Nadejda Tsankova, Frédéric Leblond, Constantinos Hadjipanayis, Kevin Petrecca

**Affiliations:** 1 Polytechnique Montréal, Montreal, Canada; 2 Centre de Recherche du Centre Hospitalier de l’Université de Montréal, Montreal, Canada; 3 Division of Neuropathology, Department of Pathology, Montreal Neurological Institute-Hospital, McGill University, Montreal, Canada; 4 Reveal Surgical, Montreal, Canada; 5 Mount Sinai Hospital, New York, NY USA; 6 Institut du Cancer de Montréal, Montreal, Canada; 7 University of Pittsburgh Medical Center, Pittsburgh, PA USA; 8 Montreal Neurological Institute-Hospital, McGill University, Montreal, Canada

**Keywords:** Biophysics, Cancer, Translational research, Optics and photonics, Optical spectroscopy

## Abstract

Safe and effective brain tumor surgery aims to remove tumor tissue, not non-tumoral brain. This is a challenge since tumor cells are often not visually distinguishable from peritumoral brain during surgery. To address this, we conducted a multicenter study testing whether the Sentry System could distinguish the three most common types of brain tumors from brain tissue in a label-free manner. The Sentry System is a new real time, in situ brain tumor detection device that merges Raman spectroscopy with machine learning tissue classifiers. Nine hundred and seventy-six in situ spectroscopy measurements and colocalized tissue specimens were acquired from 67 patients undergoing surgery for glioblastoma, brain metastases, or meningioma to assess tumor classification. The device achieved diagnostic accuracies of 91% for glioblastoma, 97% for brain metastases, and 96% for meningiomas. These data show that the Sentry System discriminated tumor containing tissue from non-tumoral brain in real time and prior to resection.

## Introduction

Together, glioblastoma, brain metastases, and meningiomas, account for nearly all intra-cranial brain tumors. Life-expectancy of patients with glioblastoma, brain metastases, and meningioma negatively correlates with the volume of tumor remaining after surgery. Time from surgery to tumor recurrence, termed progression free survival, also decreases with increasing remaining tumor volume^1–3^. While maximal resection is the goal of surgery, differentiating between tumor tissue and the surrounding brain is a challenge. For example, in cases where surgeons believed complete resection of contrast-enhancing glioblastoma bulk tumors was possible, it was achieved only one third of the time^4^. Furthermore, resections that extend into the adjacent brain can lead to neurological deficits, worsening patients’ quality of life^5–7^ and overall survival. These negative outcomes can be mitigated by the development of surgical tools that distinguish tumor tissue from surrounding brain tissue in real time during surgery and prior to tissue resection.

**Figure 1:**
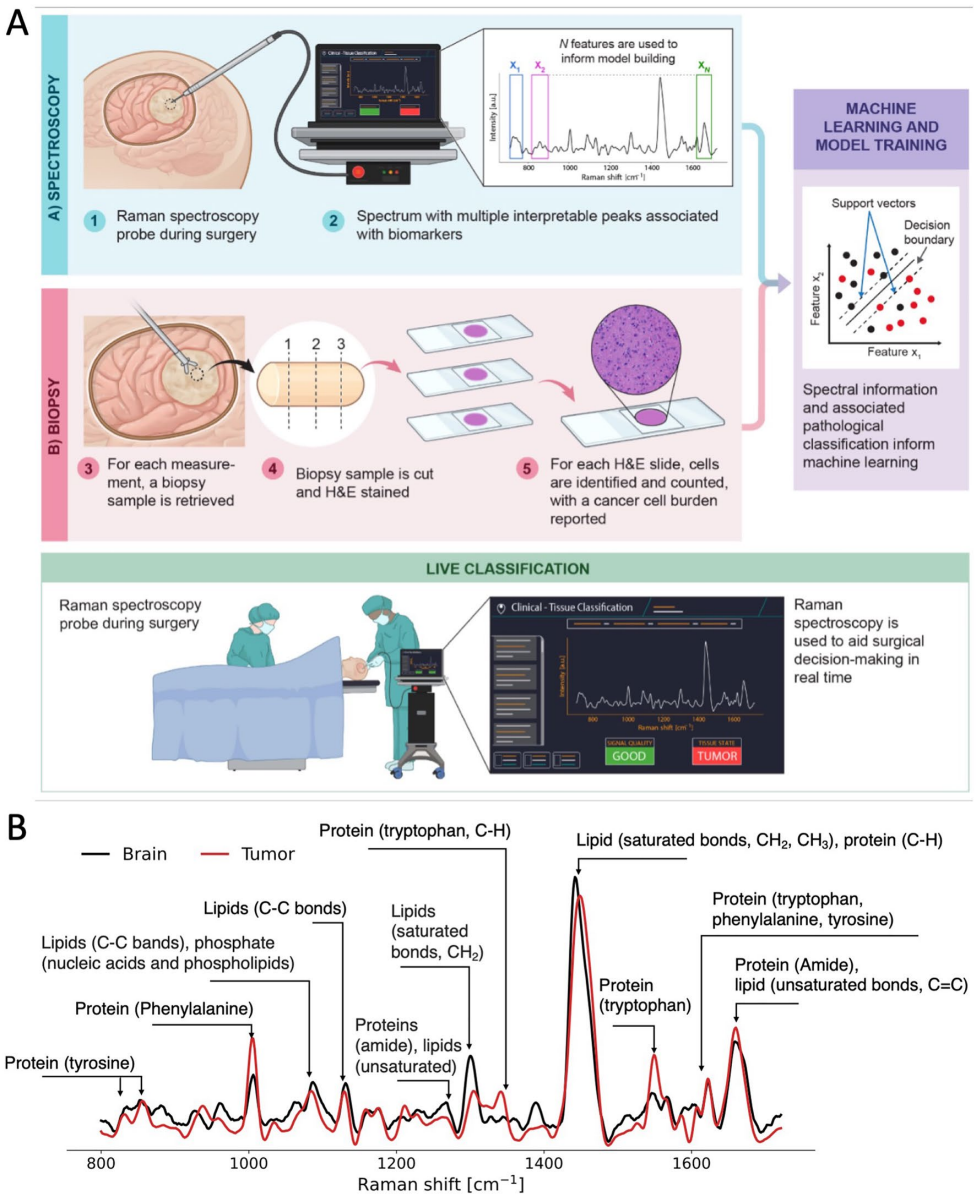
(**A**) Experimental workflow for brain tumor detection using the Sentry System. The blue panel shows spectral fingerprint measurements being acquired using the hand-held probe during neurosurgery. The red panel shows the workflow for acquisition of histopathology data associated with each spectral measurement, including estimation of cancer cell burden by the pathologist. Bulk tumor is defined as a > 90% cancer cell burden and non-tumoral brain is a cancer cell burden of 0%. The green panel shows use of the Sentry System for live classification of tumor and non-tumoral brain tissue. (**B**) Mean spectral fingerprint measurements from 67 patients showing key spectral peaks used for tumor detection. Spectral fingerprints were taken from tumor (red) and non-tumoral brain (black). C-H, carbon-hydrogen single bonds; C=C, carbon–carbon double bonds (unsaturated); C–C, carbon–carbon bonds; CH_2_, ethyl group; CH_3_, methyl group.

The Sentry System is a surgical device developed to address this clinical need. It is a hand-held tool that combines low-powered laser light in situ Raman spectroscopy measurements with machine learning to aid identification of cancer tissue. The Sentry achieves this in a label-free manner. In other words, cancer detection is achieved in a manner that is free from reagents or exogenous “labelling” compounds such as targeted fluorophores or contrast agents^8^. The hand-held portion of the device, similar in size to microneurosurgical tools, is applied to the tissue surface in question and once the in situ spectrum is acquired, the classification result, in the form of a tumor versus normal brain prediction, is displayed in real time (Fig. 1A). Here, we present the results of a multicenter study testing whether the Sentry System could distinguish the three most common types of brain tumors from brain tissue in a label-free manner.

## Methods

### Study design

This study investigated the use of the Sentry Raman spectroscopy System for intraoperative use in 67 adult patients undergoing open brain surgery at the Montreal Neurological Institute-Hospital (MNI-H, Montreal, Canada) and Mount Sinai Hospital (MSH, New York, USA). The cohort included patients with glioblastoma, metastatic cancer, and meningioma (Table 1). Forty-nine patients were recruited from the MNI-H and 18 from MSH. The study was approved by the Ethics Review Boards at MNI-H (ODS Sentry System-1000/2019-5313) and MSH (HS #: STUDY-20-01371), and informed consent was obtained from all subjects. The methods were carried out in accordance with the approved guidelines and regulations. Standard clinical imaging prior to surgery by magnetic resonance imaging (MRI) was followed, as well as a complete preoperative neurologic examination. The surgeons were blinded to information about the in situ spectral fingerprint measurements acquired during surgery.

**Table 1:** Summary of clinical characteristics of the cohorts from the Montreal Neurological Institute- Hospital (MNI-H) and Mount Sinai Hospital (MSH).

	MNI-H	MSH
Number of patients	51	19
Median age (std. dev.)	63 (11)	65 (7)
Male/Female	32/19	7/12
Brain cancer		
Glioblastoma	26	4
Meningioma		
Grade I	10	2
Grade II	2	2
Metastatic		
Lung cancer	8	4
Melanoma	2	1
Breast cancer	1	4
Kidney cancer	1	–
Colon cancer	–	1
Prostate cancer	–	1
Endometrial cancer	1	–

### Handheld Raman spectroscopic probe

The Sentry System from Reveal Surgical (Montreal, Canada) was used. It was composed of a handheld probe connected to a near-infrared (NIR) laser and a spectrometer through a fibre optic cable of length 3 m. The probe was sterilizable, reusable and had the shape of a stylet of length 12 cm. There is a conical tip of outer diameter 2.1 mm where the instrument contacts the tissue. The probe contains 9 light detection optical fibres that are circumferentially distributed around one optical fibre dedicated to tissue laser excitation. A lens at the tip of the probe ensured that both the laser spot size on the tissue surface and the area viewed through the detection fibres had a diameter of 500 µm. The excitation fibre was connected to a NIR spectrum-stabilized continuous-wave laser emitting at 785 nm with a maximum power of 350 mW (Innovative Photonic Solutions, Plainsboro, NJ, USA). Light scattered within the tissue and re-emitted from its surface was detected using a charge-coupled device (CCD) sensor (Newton model, Andor Technology, Belfast, UK) through a spectrometer slit of width 100 µm and a volume phase diffraction transmission holographic grating (Emvision LLC, model EM-VPHG-50.8-6002). The sensor was pre-cooled to − 80 °C before being used in surgery. Each spectrum acquired with the system covered a range of spectral shifts from 400 to 2000 cm^−1^, with a spectral resolution of approximately 1.8 cm^−1^. A preliminary laboratory version of the instrument from which the Sentry System was designed has been described in Jermyn et al.^8^.

### Raman spectral acquisition and intraoperative workflow

The probe was steam sterilised prior to intraoperative use and spectral fingerprint detection. An average of 30 spectra (minimum number: 1, maximum number: 80, standard deviation: 15) were acquired during each neurosurgical procedure (Fig. 1A). The number of spectral fingerprints collected for each patient is also shown graphically as individual dots in Fig. 3A, where each band of a different colour (either grey or white) represents a different patient. Region-of-interest selection for each measurement was based on pre-operative information from magnetic resonance imaging (MRI) and visual assessment by the surgeon using a surgical microscope (OPMI Pentero or Kinevo model, Zeiss, Germany). The study design ensured the number of measurements made in tumor and non-tumoral brain was balanced. During brain tumor surgery, it is common to remove non-pathological brain as part of the tumor resection. In this study, that non-pathological brain was interrogated prior to resection.

For each spectral fingerprint acquisition, the probe was placed in direct contact with the tissue and the light source of the surgical microscope was momentarily turned off. Each spectrum consisted of 20 successive spectra (repeat measurements at the same location) that were averaged to increase the signal-to-noise ratio. Each successive spectrum was obtained with a laser power of 75 mW at the probe tip with a 100 ms acquisition time.

### Histopathology analyses and sample classification

Biopsies were taken as part of the normal operating procedure. Every biopsied region had an accompanying Raman measurement taken from that region prior to biopsy. Gold standard tumor diagnosis accompanied every Raman measurement. Biopsy samples had the shape of a cylinder, with an approximate diameter of 0.5 mm and a height that was approximately 3 mm. The penetration depth of the Raman measurements is approximately 500 µm. Sample was fixed in formalin, embedded in paraffin, and sectioned prior to deposition onto a glass slide. Sections were stained with haematoxylin and eosin (H&E) and analysed by and expert neuropathologist^9^. Multiple sections of each sample were analysed to ensure tissue homogeneity throughout the sample. Example specimens are shown in Fig. S1. Samples used in this study were those classified as either tumor, if they contained only bulk tumor, defined as a > 90% cancer cell burden, or non-tumoral brain, if no tumor cells were present (i.e. a cancer cell burden of 0%). 668 were tumor and 661 samples were non-tumoral brain (Table 2). From bulk tumor and non-tumor tissue, 541 spectral fingerprints were acquired in patients with glioblastoma (518 at MNI-H, 23 at MSH), 313 in patients with metastatic cancer (243 at MNI-H, 70 at MSH) and 475 in patients with meningioma (446 at MNI-H, 29 at MSH).

**Table 2:** Pathological classification and number of spectral fingerprint measurements for each tumor type (WHO: World Health Organization; MNI: Montreal Neurological Institute-Hospital; MSH: Mount Sinai Hospital).

Diagnosis	WHO Grade	Cancer subtype	MNI-H		MSH	
			Brain	Tumor	Brain	Tumor
Brain cancer	Grade IV	Glioblastoma	175 (104)	343 (249)	8 (3)	15 (12)
Meningioma	Grade I		192 (100)	158 (158)	7 (7)	4 (4)
	Grade II		76 (57)	20 (20)	9 (9)	9 (9)
Lung cancer		Metastatic adenocarcinoma	72 (57)	30 (28)	4 (3)	2 (2)
		Metastatic carcinoma	28 (23)	11 (8)	18 (7)	7 (7)
Breast cancer		Metastatic carcinoma	–	19 (17)	9 (8)	3 (3)
Melanoma		Metastatic carcinoma	19 (17)	18 (18)	–	–
		Metastatic melanoma	19 (2)	–	8 (6)	–
Kidney cancer		Metastatic carcinoma	–	9 (9)	–	–
Colon cancer		Metastatic adenocarcinoma	–	–	4 (4)	3 (3)
Prostate cancer		Metastatic adenocarcinoma	–	–	5 (3)	7 (6)
Endometrial cancer		Metastatic carcinoma	8 (7)	10 (6)	–	–
Total			589 (367)	618 (513)	72 (50)	50 (46)

Numbers in parentheses represent the samples that remain after applying a spectral quality factor cutoff.

### Power studies

A power analysis was conducted to estimate the number of samples required to determine the likelihood that basic statistical tests (e.g., t-test) could find a statistically significant difference between non-tumoral brain and tumor tissue (either glioblastoma, metastasis, or meningioma). The software G*Power was used to perform the analysis^10^. The computation was based on a moderate effect size of 0.5 which is consistent with prior Raman spectroscopy studies^11,12^. The effect size was computed based on the average and standard deviation associated with the Raman bands at 1441 cm^−1^ (lipids and proteins) and 1004 cm^−1^ (phenylalanine).The computation revealed that the development of two-class models (e.g., non-tumoral brain versus glioblastoma) required 100 measurements per category for a statistical power of 1−β = 95% and a value α of 0.05, where β and α are Type I and Type II errors, respectively.

A posteriori analysis of the data presented in this manuscript led to an effect size > 1.8 for the models associated with specific pathologies and 1.12 for the models discriminating non-tumoral brain from tumors of any kind. All models trained/validated and tested in this study were associated with more than 100 samples per category, effectively guaranteeing a statistical confidence > 95% in our ability to reject the null hypothesis, namely that the spectral fingerprints associated with non-tumoral measurements are different than the measurements made in tumor tissue.

### Spectral fingerprint measurements and intraoperative workflow

#### Spectral pre-processing

For data analysis, Python 3.7.10 with Scikit-Learn 1.0.2 were used. Code repository for spectral pre-processing is publicly available in the paper "Open-sourced Raman spectroscopy data processing package implementing a novel baseline removal algorithm validated from multiple datasets acquired in human tissue and biofluids" Sheehy et al., *Journal of Biomedical Optics*, 28 (2), 025002 (2023)^13^ and also on Github (https://github.com/mr-sheg/orpl).

The following standard data pre-processing steps were applied to each spectroscopic measurement (Fig. S2)^14^: (1) subtraction of a ‘dark count’ background measurement acquired with the laser turned off prior to each repeat acquisition (i.e., laser-off background), (2) removal of cosmic ray events, (3) truncation of pixels with lower Raman scattering photonic counts (400–800 cm^−1^, all wavenumbers above 1750 cm^−1^), resulting in a spectrum with 521 spectral bins, (4) *x*-axis calibration using the known positions of Raman peaks from a reference material (polycarbonate resin sample^15^), (5) instrument response correction from spectral measurements acquired from a calibration material (NIST 785 nm Raman standard), (6) averaging of 20 successive measurements acquired at the same location, (7) baseline subtraction using the BubbleFill algorithm^16^ with a minimum ‘bubble’ diameter of 60 cm^−1^, (8) curve smoothing using a Savitzky-Golay filter of order 3 with a window size of 11 and (9) standard normal variate (SNV) normalization.

The BubbleFill algorithm is an iterative procedure that grows ‘bubbles’ with a diameter ranging from the full spectrum’s length up to a pre-set minimum size^13^. The diameter is expressed in wavenumber units (cm^−1^). To avoid user bias and ensure the pre-processing process could be automatically applied uniformly to the whole dataset (prior to machine learning), no fine tuning of the threshold minimum size was done. Rather, it was pre-set to correspond to the width in cm^−1^ of a Raman band ubiquitously observed in all collected Raman spectra, namely the lipid/protein band around 1441 cm^−1^. This methodological aspect of the study may explain differences in band ratios when comparing the spectra in this study with other Raman spectroscopy work studying brain^17,18^.

#### Spectral quality factor

A spectral quality factor (QF) metric was computed for each SNV-normalized spectral fingerprint. It consisted of a number of maximum value 1 quantifying the likelihood the signal was associated with a random probability distribution^16^. A random signal would have had a value of QF close to 0 while signals containing Raman spectroscopy (inelastic scattering) information were associated with QF > 0. Lower QF measurements were associated with lower inelastic scattering photonic counts and higher levels of stochastic noise, reducing their ability to reliably capture the spectral fingerprint of the tissue. The quality factor (QF) metric used in this work was defined as the average signed squared intensity^13^:

$${\text{QF}}: = \frac{1}{N}\sum\limits_{i = 1}^{N} {sgn(r_{i} )} \cdot r_{i}^{2} ,$$

where *r* is an SNV-normalized Raman spectrum and *sgn(x)* is the sign function of *x*, returning − 1 or 1 depending on whether *x* is negative or positive, respectively. Examples of individual spectra (i.e., one location in the brain for one patient) are shown corresponding to a low QF value (Fig. S2E) and a high QF value (Fig. S2F). The QF value of all spectral fingerprints acquired as part of this study are shown (Fig. 3A) along with the actual individual spectra for non-tumoral and tumor samples, in the form of spectrograms (Fig. 3B, C). To determine the optimal QF, receiver operating curves (ROCs) were made with different QF thresholds. The final QF cut was the one with the best area under the curve (AUC) that does not lead to imbalanced datasets towards either class.

#### Machine learning models

Machine learning models were developed for the detection of glioblastoma, metastatic cancer, or meningioma, and one all-encompassing tumor detection model was developed from all measurements, independent of tumor type (Fig. 4). Each of the four classification models was developed from a training set composed of 80% of the spectral fingerprint measurements from the MNI-H and MSH (Fig. S3). For each model, a testing set (i.e., holdout set) associated with the remaining 20% of all spectral fingerprint measurements was held out to evaluate the performance of the models on an independent dataset. The constitution of the testing sets was such that they had approximately the same percentage of samples from MNH-H and MSH patients as in the training sets. All samples from a given patient were either in the training or the holdout set, to remove potential biases arising from sharing patient samples between the training/validation and testing phases.

Prior to machine learning model training/validation and testing, a Gaussian fitting technique was applied to each spectral fingerprint measurement that was described in Plante et al.^19^. Briefly, this technique fitted a Gaussian function on any peak with a prominence of 0.1, a height of 0.5 (relative to the lowest value in the SNV-normalized spectrum), and a tolerance of ± 2 cm^−1^ on the position of the peak, considering that the Raman spectrum intensity ranges from − 2 to 7 in normalized intensity (SNV normalisation). Only the peaks that were present in 50% of all measurements were retained as potential features^19^. This procedure extracted the position in wavenumbers, the height, and the width of up to 11 different peaks. The specific number of peaks retained depended on the pathology type, i.e., on which machine learning model was trained. The height and width of those peaks (up to 22 variables in total)—herein labelled the *peak features*—along with the relative intensity of the 521 individual bands within each spectrum, constituted the set of potential spectral features from which machine learning models could be trained. Prior to model training/validation and testing, the number of features was reduced to include only those that contributed the most to the variance between non-tumoral brain and tumor. This feature selection step was accomplished using a random forest algorithm with 200 estimators where the maximum number of features (*N*) was the only floating hyperparameter^20^. This technique was used by our group in multiple Raman spectroscopy publications, both for cancer detection in tissue^21^ and for biofluid interrogation to detect COVID-19 infection^22^. The feature selection process is essentially a dimensional reduction step implemented prior to machine learning model training/validation and testing. A different method that is commonly used by other Raman spectroscopy groups is principal component analysis (PCA)^23^.

Machine learning model training from the dimensionally-reduced features set was done using linear SVM with the regularization parameter *C*. Unbalanced classes in each model are accounted for with a class weight parameter adjusted to reflect the ratio between non-tumoral and tumoral brain samples^21,24^. Each time a model was trained, hyperparameters were selected by carrying out a grid search across many combinations (*N, C*). The regularization parameter *C* was varied between 0.01 and 5, the number of individual band features was varied between 5 and 25 and the number of *peak features* varied between 2 and 20, such that *N* (i.e., the total number of features) ranged between 7 and 45. For each combination, performance was assessed using five-fold cross validation based on the number of false/true positives and false/true negatives, by comparing the model prediction with the assigned pathological label (tumor or non-tumoral brain). Specifically, the training dataset was split into five non-overlapping subsets (folds). Each fold consisted in training a model from 4 of the 5 subsets, while the remaining subset (validation set) was used to assess performance. This resulted in one set of hyperparameters (*N, C*) (i.e., a model) that minimized the number of false positive and false negative predictions. The final model was applied to the holdout data subset and performances were reported as a receiver operating characteristic (ROC) analysis. Accuracy, sensitivity, and specificity were calculated from the ROC curve and the ROC curve area under curve (AUC) was reported. The region between 1500 and 1620 cm^−1^ was removed in the feature selection as this region can be associated with peaks due to haemoglobin.

Two sets of predictive models were developed, one set without any QF threshold (i.e., no spectral quality cutoff) applied to the spectral fingerprint data and one keeping only higher quality data. Models with no QF threshold consisted of (1) 183 non-tumoral brain and 358 glioblastoma samples, (2) 194 non-tumoral brain and 119 metastasis samples, (3) 284 non-tumoral brain and 191 meningioma samples, and (4) a total of 661 non-tumoral brain and 668 tumor samples (Fig. S4). Higher quality models consisted of (1) 107 non-tumoral brain and 261 glioblastoma samples, (2) 137 non-tumoral brain and 107 metastases samples, (3) 173 non-tumoral brain and 191 meningioma samples, and (4) a total of 417 non-tumoral brain and 559 tumor samples (Figs. 4 and S3). The higher quality dataset consisted of spectra with QF > 0.5 for glioblastoma and metastatic patients and QF > 0.3 for meningioma patients. Processing and classifier results can be obtained in less than 0.1 s, achieving real-time classification when implemented in the clinic.

### Ethical compliance statement

Institutional Review Board Protocols from McGill University Health Centre and Neurological Institute (ODS Sentry System-1000/2019-5313) and Mount Sinai School of Medicine (HS #: STUDY-20-01371) were approved for the collection and use of human brain tissue specimens, corresponding histology images and Raman spectra. Informed consent was obtained from all participants and methods were carried out in accordance with the approved guidelines and regulations.

## Results and discussion

**Figure 2:**
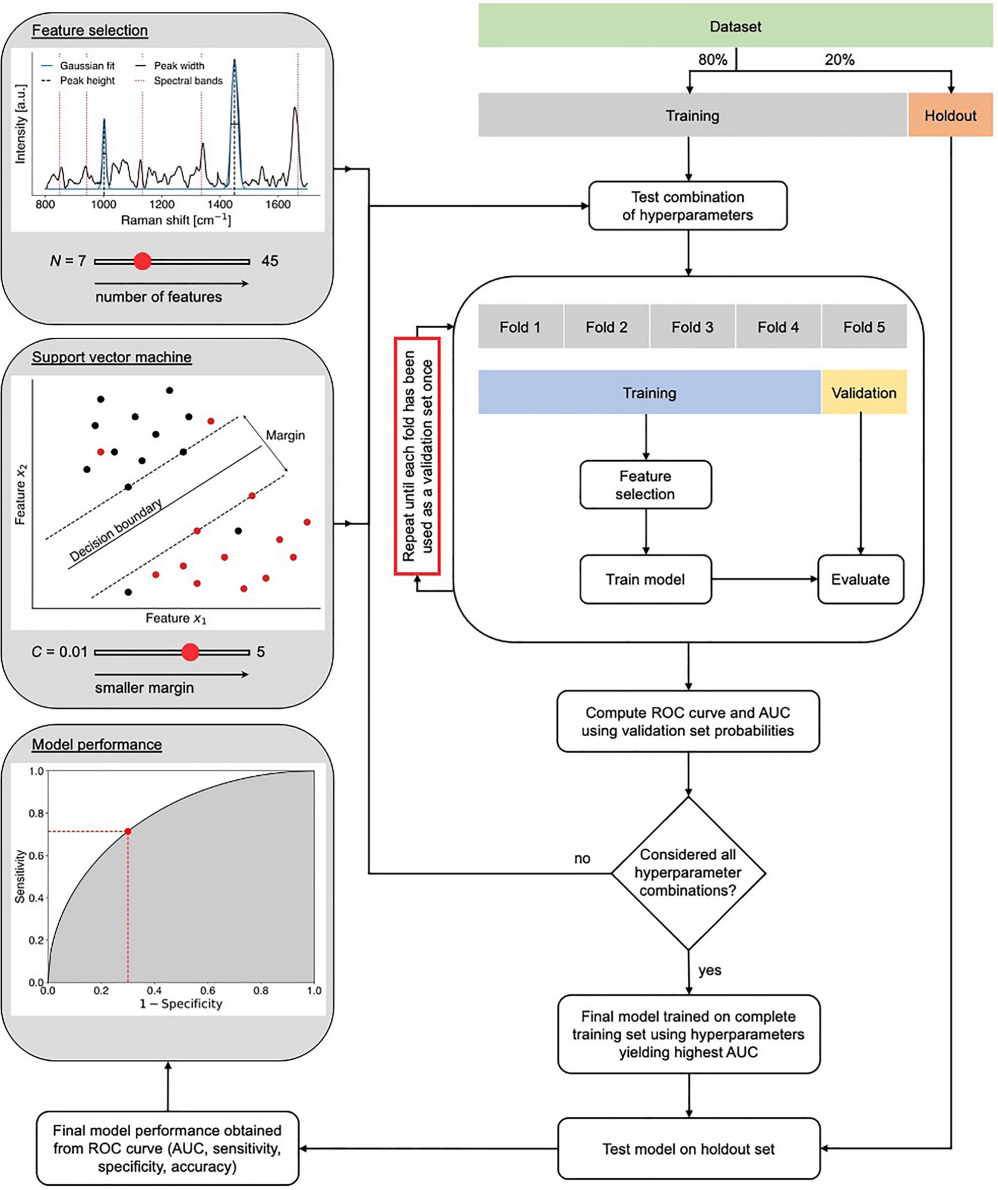
Schematic diagram of the machine learning workflow. The dataset was split into training (80% of the whole dataset) and holdout (20% of dataset) subsets. Feature selection and classification hyperparameters were optimized by generating machine learning models using support vector machines (SVM) for all predefined combinations of the hyperparameters *N* and *C*. The model performance associated with each combination was assessed using a fivefold cross-validation technique based on ROC analyses comparing model predictions with the assigned pathology labels. The final model was trained on the complete training set using the hyperparameters that yielded the lowest number of false positives and false negatives.

The Sentry System uses machine learning models that were developed based on support vector machines (SVM) for each tumor type by correlating the intraoperative spectral acquisition with gold-standard pathological analysis for each sample (Fig. 1A). It is the training, validation, and testing on independent data of these models that is presented here (Fig. 2). Spectral peaks are informed by the biomolecular content of the tissue at the interrogated site, and the peak height provides information about the relative concentration of these molecules (Fig. 1B)^25^. Biomolecular structures that are sensed in the brain include the amide backbone of proteins, aromatic amino acids (phenylalanine, tyrosine, tryptophan)^22,26,27^, and lipids^28^ (Fig. 1B). In this study, a sample was designated as tumor if it contained only bulk tumor, or non-tumoral brain if it did not contain tumor cells (“Methods”). The spectral data subset for non-tumoral brain is associated with an approximately equal fraction of samples that were either pure normal grey matter, pure normal white matter, or a mix of white and grey matter.

**Figure 3:**
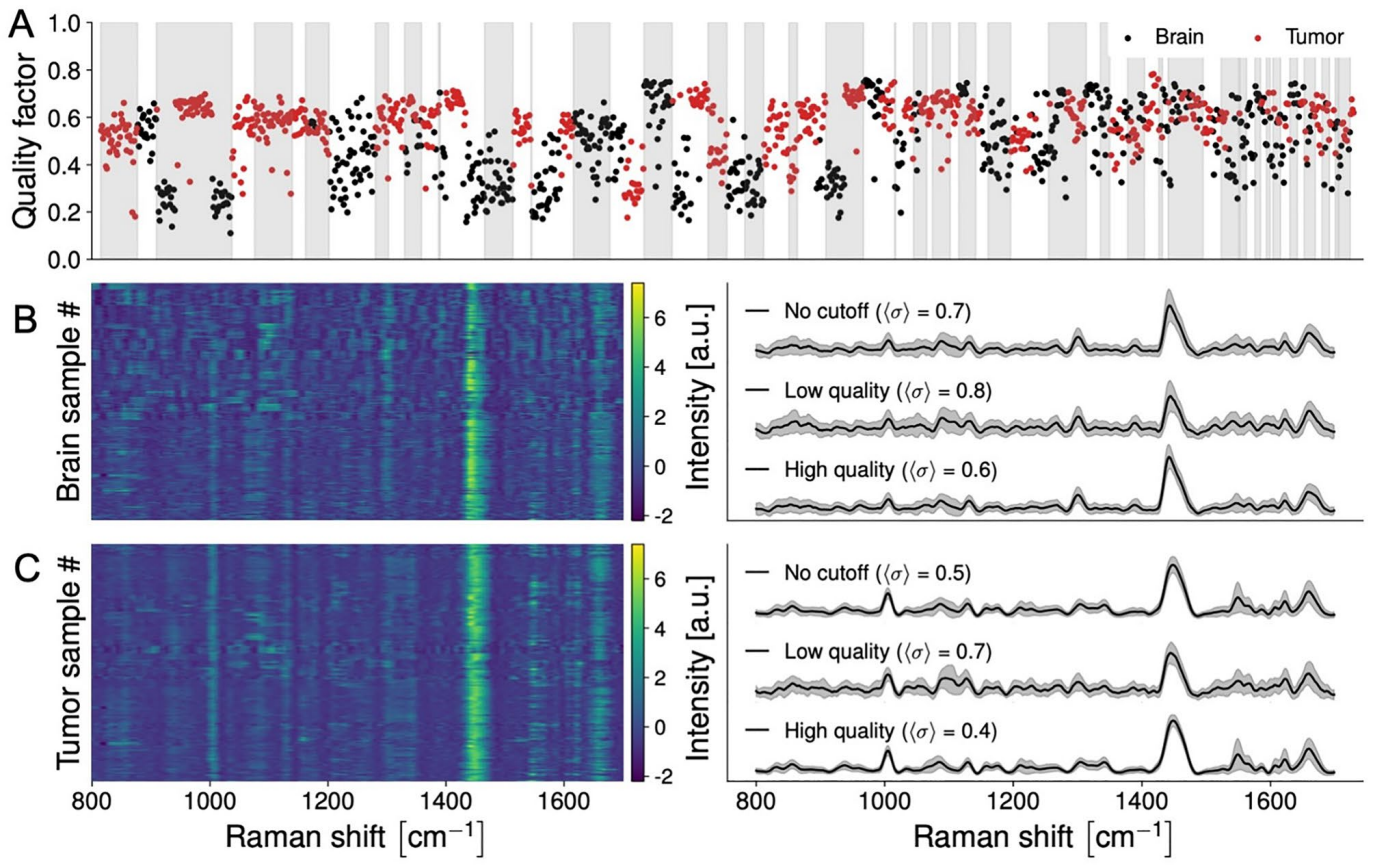
(**A**–**C**) Depiction of the spectral quality factors for brain and tumor samples acquired with the Sentry system. (**A**) Quality factor (QF) distribution of all Raman spectra with alternating grey and white bands denoting different patients. (**B**) Spectrogram of Raman spectra from non-tumoral brain (left) and average Raman spectra with their variance (right). Average spectral fingerprints are shown for all samples (no QF cutoff) as well as for high and lower quality spectra. (**C**) Spectrogram of Raman spectra from tumor samples (left) and average Raman spectra with their variance (right). Higher quality spectra are associated with smaller levels of stochastic (photonic) noise leading to smaller inter-measurement variances (shown by sigma values).

**Figure 4:**
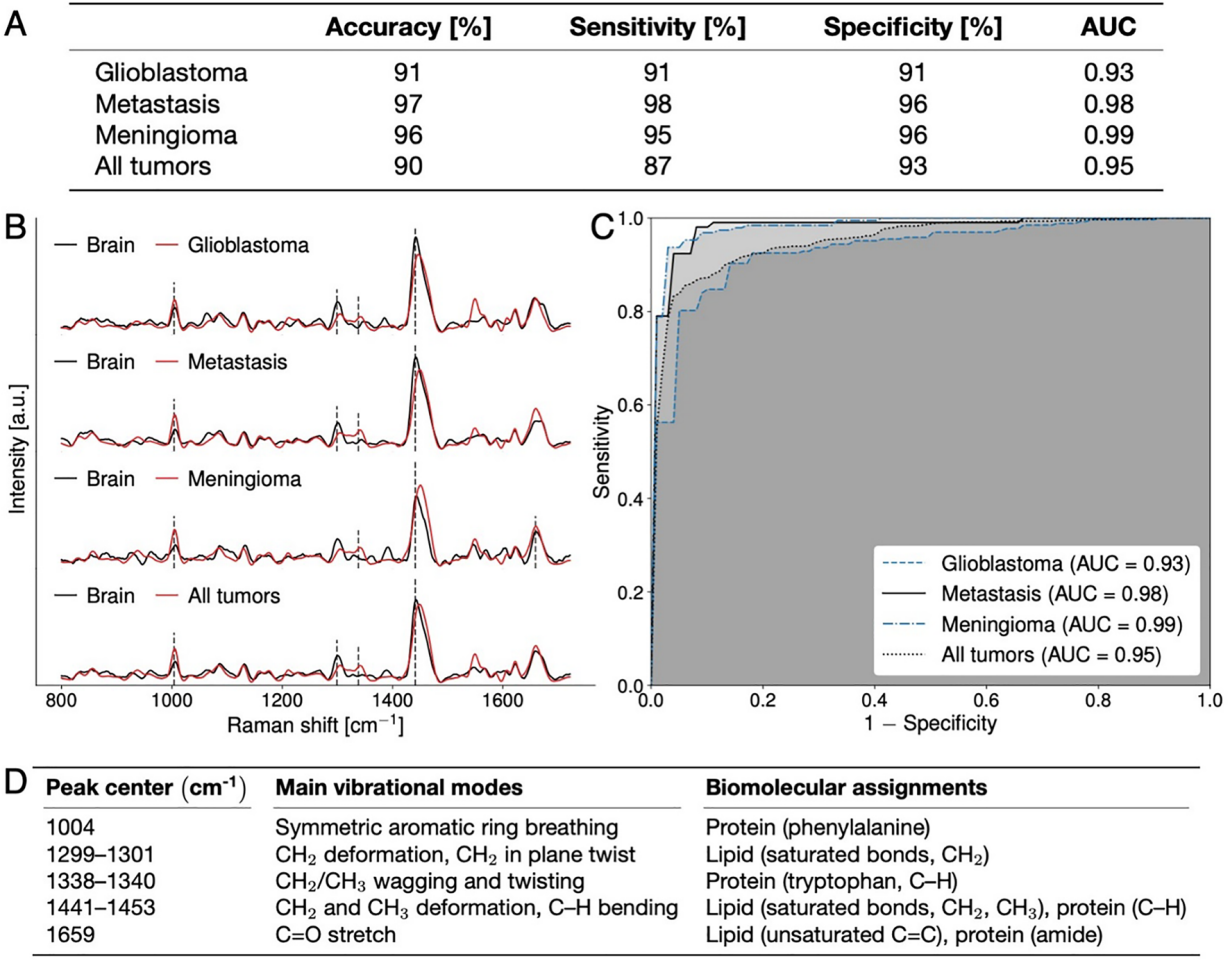
(**A**–**D**) Machine learning models discriminating between spectral fingerprints from non-tumoral brain and bulk tumor for glioblastoma, metastasis, meningioma and all tumors using data from Montreal Neurological Institute Hospital (MNI-H) and Mount Sinai Hospital (MSH). (**A**) Table plotting accuracy, sensitivity, specificity, and area under curve (AUC) for all models. (**B**) Spectral fingerprints from all patients (from MNI-H and MSH) with each specific type of brain tumor. Main spectral features used in model building designated by dotted lines, with (**D**) peak location and biomolecular origin specified. Mean non-tumoral brain spectra are shown in black and tumor spectra are shown in red. (**C**) Receiver operating curve (ROC) for the predictive model with area under curve (AUC) for each model. C–H, carbon-hydrogen single bonds; C=C, carbon–carbon double bonds (unsaturated); C–C, carbon–carbon bonds; CH_2_, ethyl group; CH_3_, methyl group. Quality factor cutoffs have been applied in all cases.

Nine hundred and seventy-six spectral measurements (559 in tumor, 417 in non-tumoral brain (Table 1)), from 67 patients (49 from Montreal Neurological Institute-Hospital, 18 from Mount Sinai Hospital) (Table 2) were included in this study. Spectra were pre-processed using standard techniques including cosmic ray removal, baseline correction, normalization, and wavenumber calibration (“Methods”, Fig. S2). The Sentry System, using tumor type-specific machine learning classification models (“Methods”), discriminated tumor from brain with > 90% sensitivity and specificity (Figs. 4, S3) across tumor types: glioblastoma versus brain with 91% sensitivity and 91% specificity (Figs. 4, S3A); metastases versus brain with 98% sensitivity and 96% specificity (Figs. 4, S3B); and meningioma versus brain with 96% sensitivity and 96% specificity (Figs. 4, S3C). Using a non-tumor type specific model, the device discriminated brain versus tumor (either glioblastoma, metastases, or meningioma) with 87% sensitivity and 93% specificity (Figs. 4, S3D). All performance metrics were obtained based on the application of the machine learning models to a hold-out testing set that was completely independent from the training/validation set (Fig. 2). The testing set was composed of data from both institutions. All spectral fingerprints collected are shown, along with a spectral quality factor assessing the ability of the system to capture the Raman spectroscopy tissue biomolecular fingerprint (Fig. 3). The machine learning models were developed after the rejection of lower-quality spectral fingerprint data by applying a cutoff, resulting in rejection of 26% of the whole dataset (originally 1341 spectra). Machine learning models were also trained/validated and tested without the application of a spectral quality factor cutoff (i.e., using the full dataset). This resulted in slightly inferior predictive performance (Fig. S4A): glioblastoma versus brain with 85% sensitivity and 85% specificity (Fig. S4B); metastases versus brain with 93% sensitivity and 92% specificity (Fig. S4C); meningioma versus brain with 97% sensitivity and 97% specificity (Fig. S4D); and non-tumor type specific versus brain with 83% sensitivity and 91% specificity (Fig. S4E).

**Figure 5:**
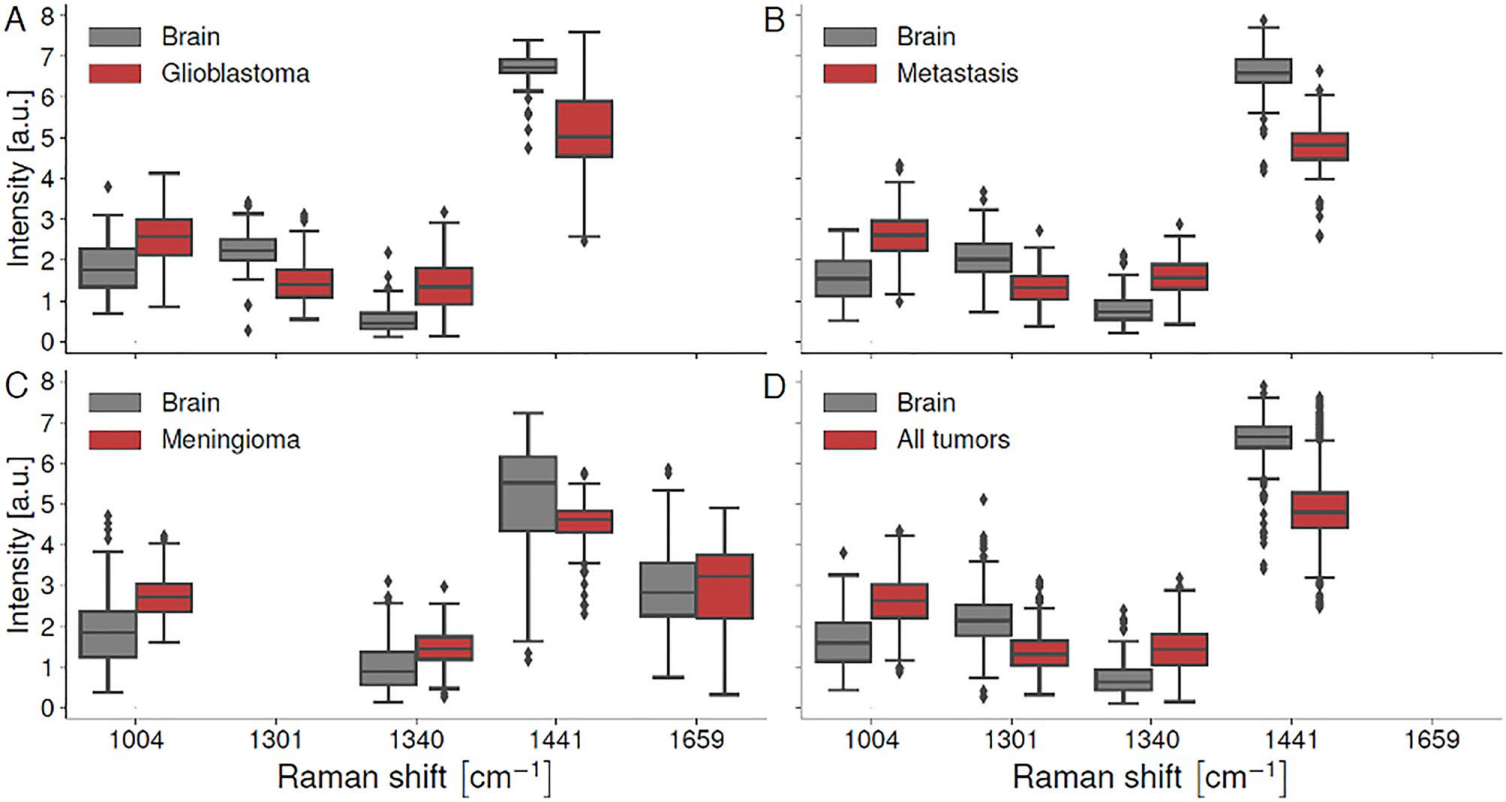
(**A**–**D**) Box and whisker plots associated with the five most important bands selected by machine learning to distinguish non-tumoral brain from tumor: (**A**) non-tumoral brain vs. glioblastoma, B) non-tumoral brain vs. metastases, (**B**) non-tumoral brain vs. meningioma, (**C**) non-tumoral brain vs. tumors (glioblastoma, metastases and meningioma measurements lumped together). *P*-values for all bands on all plots are p < 1e^−4^ except for the 1659 cm^−1^ band which had p > 0.05 (p = 0.15).

The machine learning models mainly used four biomolecular features (i.e., intensities from individual spectral bands) that separated tumor and brain spectral fingerprints (Figs. 1B, 4 and 5). These brain cancer Raman spectroscopy biomarkers are associated with protein (phenylalanine) at 1004 cm^−1^^26,27^; protein (tryptophan) at 1340 cm^−1^^10,27^; lipids at 1299 cm^−1^^28^; and the lipid and protein peak at 1441 cm^−1^^26,29^ (Fig. 5, Table S1). Increased levels of collagen in the extracellular matrix have been reported in glioblastoma^30^ and other tumors^31^, and may underlie the elevated protein contributions detected in tumors here. Lipid content was lower in tumor compared to brain, consistent with studies analyzing lipid content in tumors using brain biopsies and analytical chemistry^32^. The mean Raman spectra for tumor tissue exhibited lower variance than spectra from normal tissue (Fig. 3). This is perhaps because tumors may have similar biochemical characteristics to each other in terms of their microenvironment, such as tumor infiltrating immune cells^33,34^, increased vascularization^35^ and increased deposition of extracellular matrix components such as collagen^36^. These properties may distinguish tumors from non-tumor tissue.

Other approaches to improve brain tumor surgery include intra-operative MRI (iMRI) and fluorescence-guided surgery (FGS) with 5-aminolevulinic acid (5-ALA) for glioblastoma. Highlighting the need for intra-operative aids, Senft et al.^37^ showed that iMRI use led to additional resection of contrast-enhancing tissue in one third of patients. While it provides updated structural surgical planning imaging, iMRI is limited as it does not provide information about the nature of the surgical tissue during surgery, brain shift during the surgery remains a challenge, and the initial infrastructure and operating costs are prohibitive. 5-ALA FGS has also been shown to reduce residual contrast-enhancing glioblastoma tumor following surgery. However, it is limited in detection of metastasis, where only 66% of tumors are detected^38^, and meningioma, where there is insufficient evidence that it aids resection^39^.

Hollon et al.^40^ showed stimulated Raman histology (SRH) could be used on brain biopsy specimens to diagnose brain tumor types. This technique is a form of stain-free, deep learning-based histology that has the potential to replace frozen section histopathologic analyses. It requires that brain tissues are biopsied and processed before imaging with an SRH microscope. This technique could be helpful for tissue diagnosis but would prove difficult to implement as a real time surgical guidance tool. In contrast, the Sentry system provides real time feedback about the disease state of brain tissue in situ, prior to resection, reducing the likelihood of removing non-tumoral brain and increasing the likelihood of removing tumor. In other work, Raman spectroscopy has been reported to distinguish non-tumor and tumor brain tissue ex vivo. Bury et al., discriminated between normal brain and tumor in fresh-frozen ex vivo glioma and meningioma tissue using Raman-based microscopy^41^, and on fresh ex vivo tissue samples using gold nanoparticles and a Raman spectroscopy probe^42^.

Previous in vivo work by our group tested a prototype laboratory version of the device, preliminarily demonstrating glioma detection with 90% accuracy in 17 patients (161 spectral fingerprint measurements)^8^. Compared to this study, the machine learning model used in this previous work was not tested on an independent dataset: rather a leave-one-out cross validation technique was used. Thus, the generalizability to new patients and new data had not been demonstrated. Moreover, this current study expands beyond gliomas by including metastasis and meningiomas and it is associated with a seven-fold increase in the number of collected spectra. This last point is crucial since it allows assessment of model generalizability to new data using hold-out testing sets, ensuring intra- and inter-patient variability is accounted for (Fig. 2). The original study was also carried out with a single prototype unit used by one surgical team with no live data quality assessment, often leading to data loss. The Sentry System used in this current study incorporates hardware and signal processing advances that optimize Raman spectroscopy signal-to-noise ratio during tissue measurement, as well as reproducibility between instruments. The previous system was only suitable for use by a research team with extensive training in use of the device, while the device used here incorporates control software providing a user interface suitable for a standard surgical team. The new software can identify—in real time—poor quality data (e.g., using the spectral quality factor (Fig. 3)), ensuring that only high-quality measurements are used for tissue characterization.

Here, we provide results of the first multi-user experience using the Sentry System to detect the most common types of brain tumors label-free during surgery and in real time. Two different Sentry System units were used by different surgical teams and independently tested on data acquired at these centers. This demonstrated the new cancer detection machine learning models generalized well to new data. The device, equipped with these machine learning models, is therefore ready for deployment. It has been conceived with an engineering design ensuring consistent quality data as well as compliance with relevant industry standards and readiness for clinical translation. It performed robustly across brain tumor types allowing for a high degree of confidence for users to distinguish brain tumors from non-tumoral brain. The ease of use of this device, coupled with its high performance may improve the safety and effectiveness of brain tumor surgery, positively impacting patient outcomes.

### Supplementary Information

Supplementary Information.

## Data Availability

To obtain anonymized samples, images, or processed Raman spectra, please contact Frederic Leblond directly. Code repository for model training, analysis and validation is publicly available in the paper "Open-sourced Raman spectroscopy data processing package implementing a novel baseline removal algorithm validated from multiple datasets acquired in human tissue and biofluids" Sheehy et al.^13^*,* Journal of Biomedical Optics, (2023) and also on Github (https://github.com/mr-sheg/orpl).
