# Supplementary material for: In situ brain tumor detection using a Raman spectroscopy system—results of a multicenter study
Source: Sci Rep. 2024 Jun 10;14:13309. doi: 10.1038/s41598-024-62543-9 (PMC11164901; doi:10.1038/s41598-024-62543-9)
Supplement: Supplementary file 1 — Supplementary Information. [file 41598_2024_62543_MOESM1_ESM.docx]

**Supplementary figures and tables**

**Table S1.** Assignment of Raman-active biomolecular vibrational modes from literature. The features (peak center) assigned to “lipid” are in comparison to spectral fingerprints from purified brain lipids^45^ including phosphatidylethanolamine, phosphatidylcholine and sphingomyelin.^28^ The features assigned to “protein” are in comparison to spectral fingerprints from purified proteins such as collagen.^26^ Legend: C-H: carbon-hydrogen single bonds, C=C: carbon-carbon double bonds (unsaturated), C-C: carbon-carbon bonds, CH_2_: ethyl group, CH_3_: methyl group.

**
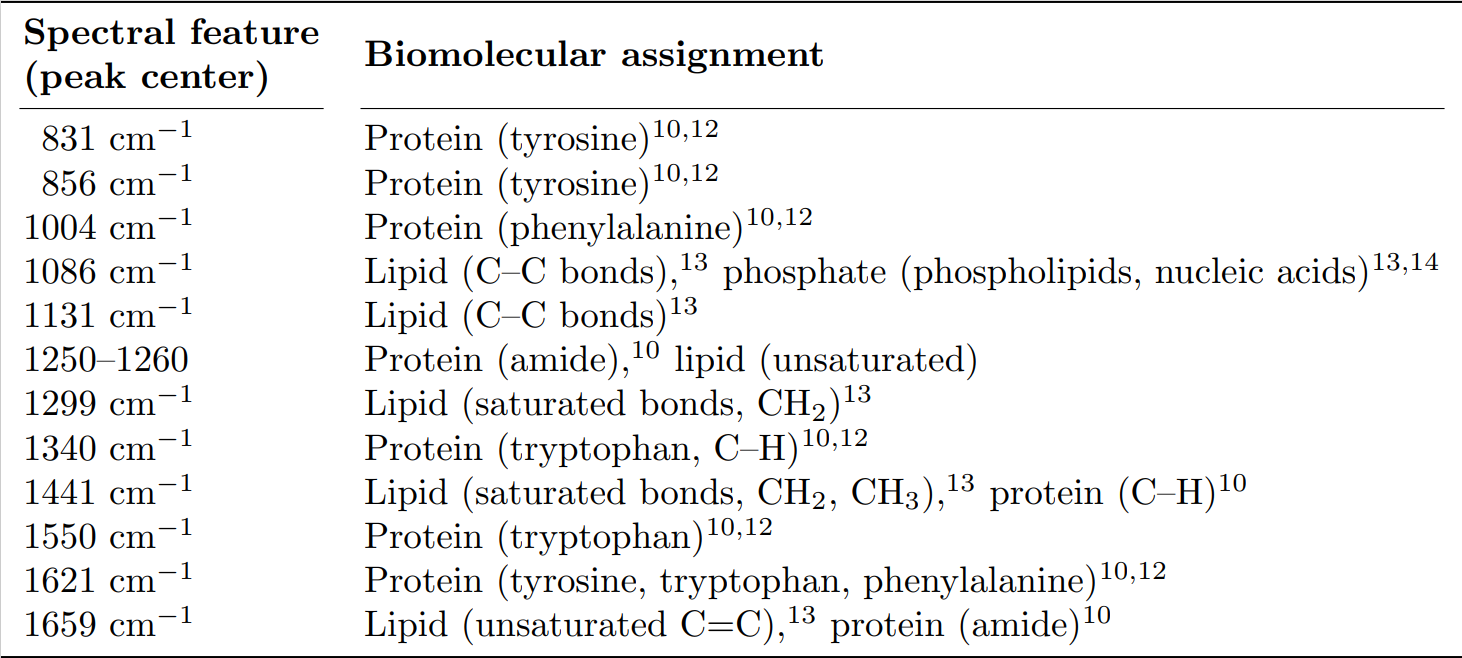
**

**
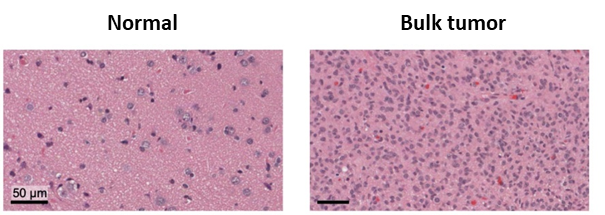
**

**Supplementary Figure S1: Haemotoxylin and eosin stains of normal and bulk tumor brain tissue sections.** “Normal brain” indicates a cancer burden of 0% and whilst a cancer burden of >90% is “tumor” tissue. The scale bar is 50 micrometers. This example is from a patient with glioblastoma.


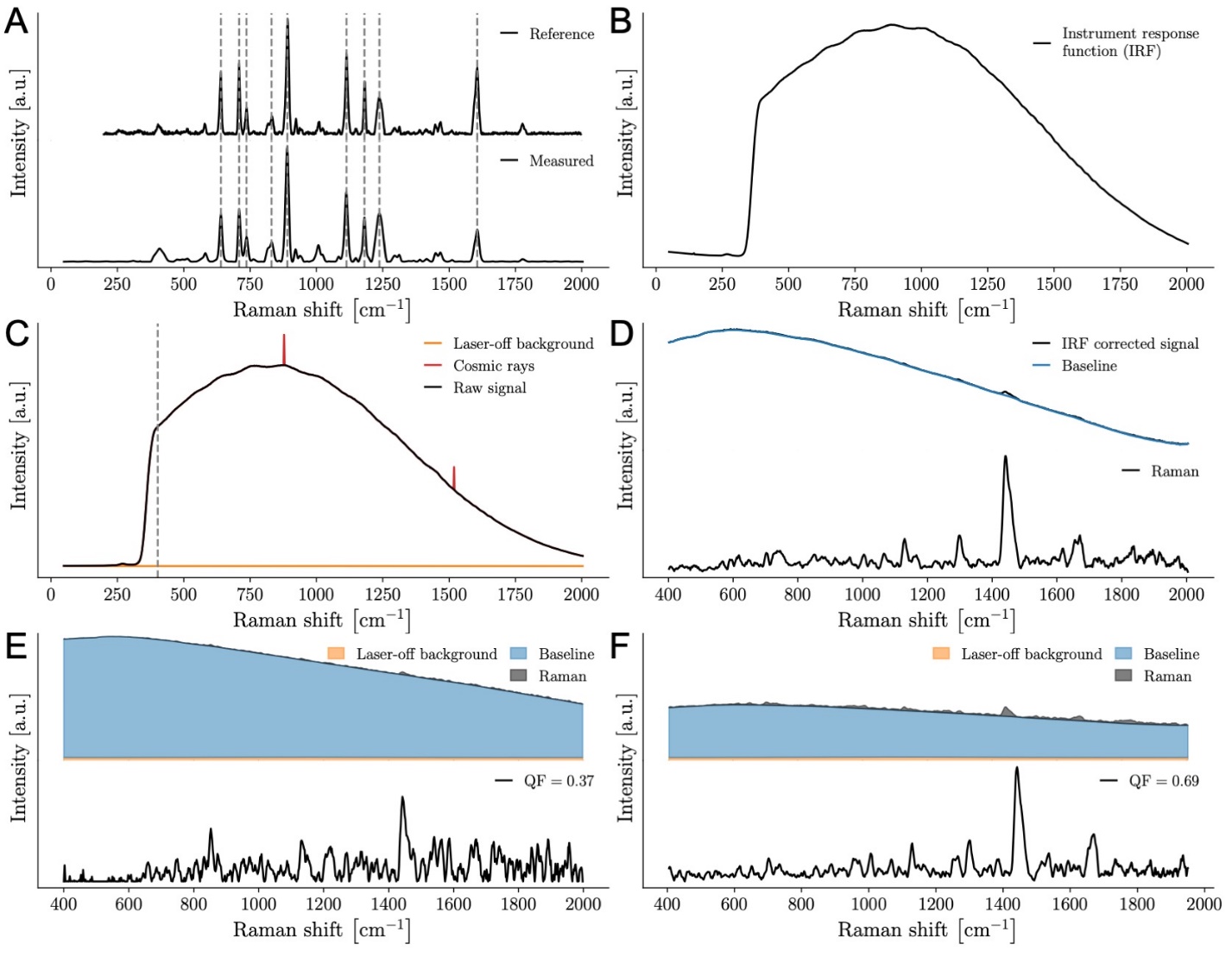


**Supplementary Figure S2: (A-F) Data processing process.** (A) Reference spectrum of polycarbonate used to convert CCD camera pixels into wavenumbers based on a polycarbonate spectrum measured with the Sentry system. (B) Instrument response function (IRF) measured with the system from the National Institute of Standards and Technology (NIST) Raman standard. (C) Dark count background acquired with the laser turned off and unprocessed (raw) *in situ* brain spectrum showing cosmic ray events. The grey dotted line designates the region that is truncated before further spectral pre-processing. (D) Spectrum after removal of cosmic rays and normalization with the IRF with the non-Raman baseline contribution isolated using the BubbleFiill shown in blue. The SNV-normalized Raman spectrum is shown at the bottom. (E-F) Representation of correction applied to (E) one low quality spectrum (QF = 0.37) and (F) one high quality spectrum (QF = 0.69).


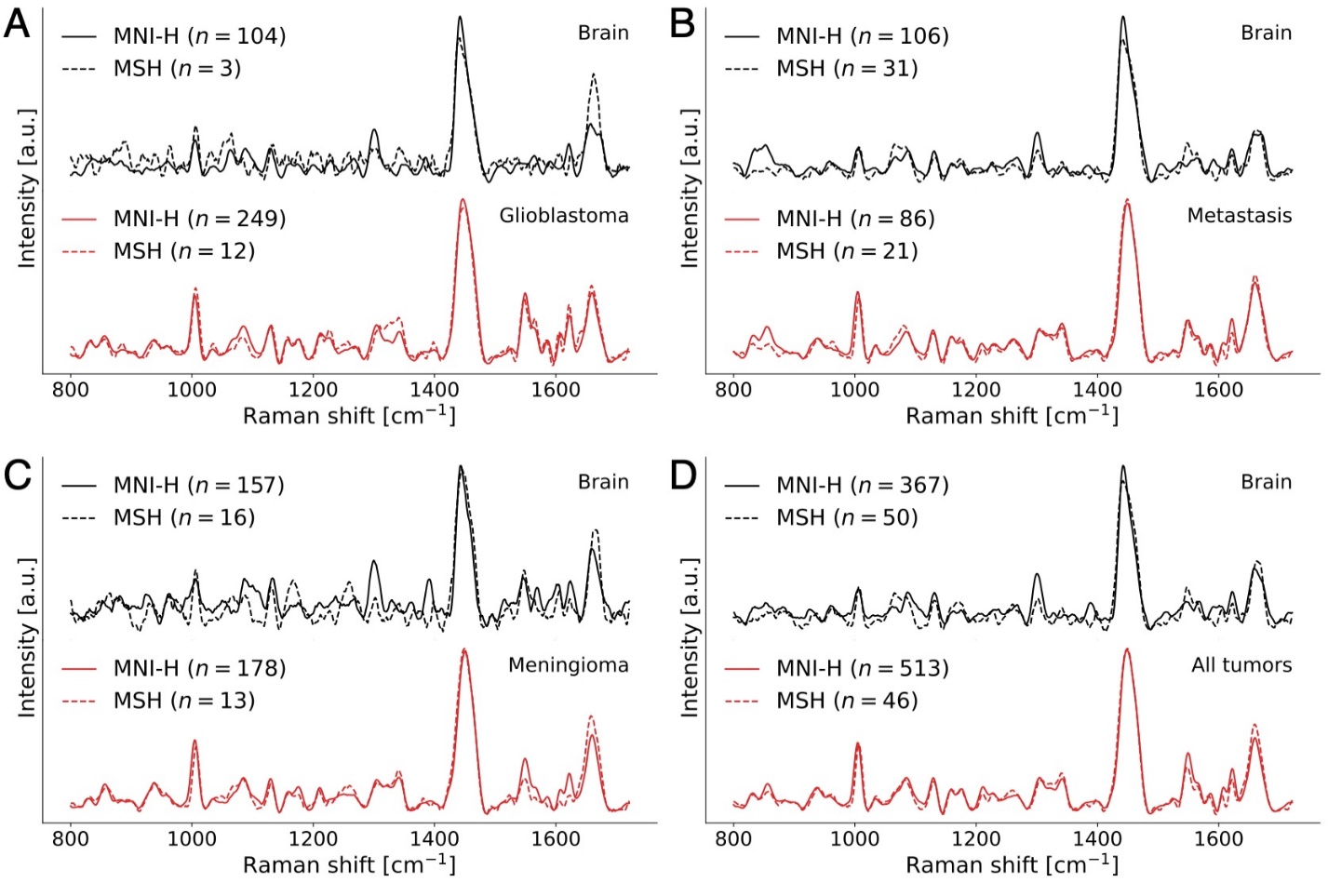


**Figure S3.** **(A-D) Data from MNI-H and MSH showing spectral fingerprints from each medical center with quality factor applied.** Spectral fingerprints were plotted for patients with (A) glioblastoma, (B) metastasis, (C) meningioma, and (D) all tumor types combined.


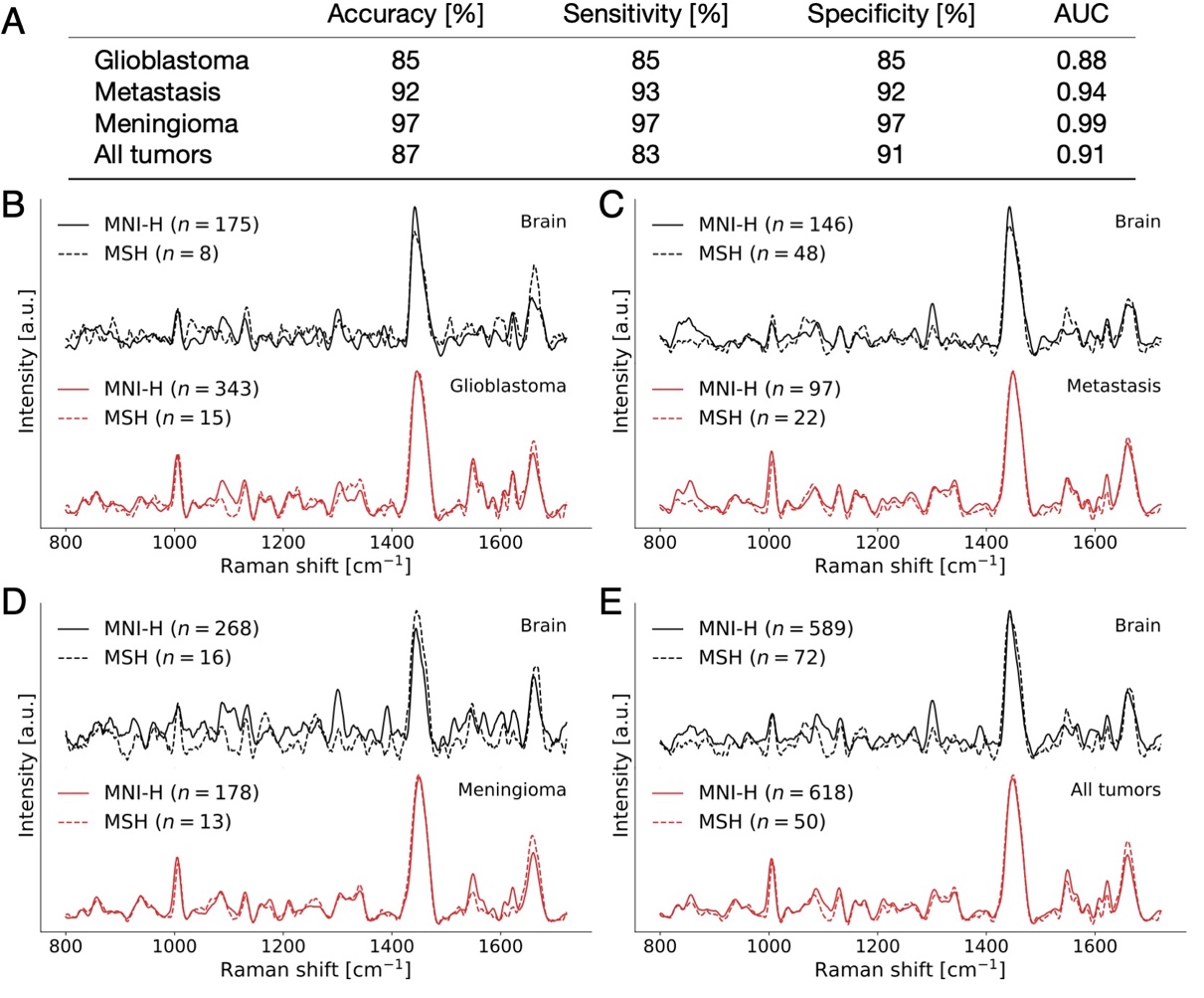


**Supplementary Figure S4: (A-E) Machine learning model with no quality factor threshold applied to the data.** (A) Table presenting all results from machine learning models discriminating between spectral fingerprints from non-tumoral brain and tumor in patients with different types of cancer. Spectral fingerprints were plotted for patients with (B) glioblastoma, (C) metastasis, (D) meningioma, and (E) all tumor types.
